# Exploring patient perspectives on Iran’s Electronic Prescription System: a Qualitative Inquiry

**DOI:** 10.3389/fmed.2024.1385256

**Published:** 2024-07-04

**Authors:** Sajed Arabian, Somayyeh Zakerabasali, Mohammad Javad Raee

**Affiliations:** ^1^Student Research Committee, Department of Health Information Management, School of Health Management and Information Sciences, Shiraz University of Medical Sciences, Shiraz, Iran; ^2^Department of Health Information Technology, Varastegan Institute for Medical Sciences, Mashhad, Iran; ^3^Clinical Education Research Center, Health Human Resources Research Center, Department of Health Information Management, School of Health Management and Information Sciences, Shiraz University of Medical Sciences, Shiraz, Iran; ^4^Department of Pharmaceutical Biotechnology, School of Pharmacy, Shiraz University of Medical Sciences, Shiraz, Iran

**Keywords:** electronic prescription, health information technology, doctor, pharmacist, qualitative analysis

## Abstract

**Background:**

Electronic prescriptions represent a fundamental shift in service delivery, healthcare management, and associated costs, offering numerous advantages. However, akin to other electronic systems, they also present challenges. This study aimed to investigate patients’ understanding of the challenges associated with electronic prescriptions in Iran.

**Methods:**

This study used a qualitative research design, utilizing individual and semi-structured interviews with patients referred to selected pharmacies across all 11 districts of Shiraz City. The data were analyzed using MAXQDA software (version 10), and descriptive statistics for demographic data were calculated using SPSS version 19.

**Results:**

The study revealed that the participants generally demonstrated a certain level of familiarity with electronic prescribing systems. However, it was evident that many were unaware of the potential implications of such technology for their relationships with healthcare providers. This underscores the urgent need for patient understanding in the context of the electronic prescription system. While patients were relatively familiar with the functionality of electronic prescribing systems, they lacked a comprehensive understanding of how using these systems could affect their interactions with healthcare providers.

**Conclusion:**

Patients are significant beneficiaries of the electronic prescribing system. By addressing their needs and concerns, they can develop a positive attitude toward this system. Their active engagement can pave the way for the system’s ease of use, increase its acceptance, and ultimately enhance the quality of healthcare services.

## Introduction

Electronic prescription, typically defined as the transition from paper prescriptions to electronic systems facilitating the creation, transmission, and processing of prescriptions by healthcare providers and pharmacies, represents a pivotal transformation in healthcare management. This includes a range of activities, from patient registration to information retrieval and service provision, all within the context of electronic systems. Many countries are transitioning toward electronic systems to improve safety, efficiency, and cost-effectiveness in healthcare delivery, with electronic prescriptions being a key component of this transformation (1–3). Electronic prescribing is intended to alleviate the burden on patients with chronic conditions, reducing the necessity for frequent consultations with their physician, as electronic prescriptions can be issued through online consultations. This approach saves both time and money for the therapist and patient, and so far, it has reduced up to 60% of chronic patients’ visits to the doctor (4, 5).

There has been a significant increase in the adoption of electronic outpatient treatment services, which is indicative of a global trend toward digitizing healthcare delivery. This shift from manual to electronic systems underscores the crucial role of digital transformation in healthcare services worldwide (6–8). In most European Union member states, healthcare services are progressively provided electronically. This trend is expected to accelerate, with initiatives to integrate these systems into an international electronic health service framework. This integrated network will enable citizens to have seamless access to medications and medical services across member countries, primarily facilitated by electronic prescribing initiatives (9–11).

Electronic prescriptions offer numerous advantages, including reducing revisits for chronic patients, ensuring accurate insurance information, minimizing prescription errors during pharmacy delivery, enabling patients to purchase items from different pharmacies with a single prescription, and providing access to prescription histories. Moreover, it eliminates paper documentation, reduces production costs, assists treatment decisions through decision support systems, and enhances service quality for patients (10, 12). Research indicates a preference for electronic prescribing among primary care practitioners, citing benefits such as enhanced legibility, reduced medication errors, and streamlined workflows (4, 8, 9, 13). Conversely, the inherent challenges of manual prescription systems, such as illegible handwriting leading to medication errors, underscore the pressing need for electronic prescribing solutions to enhance patient safety and elevate the standard of care (4, 9, 13).

However, electronic prescribing systems are not without their challenges. These include high setup, maintenance, and training costs for medical staff, bandwidth limitations leading to system outages, security and privacy concerns among users, an increase in physician errors in electronic prescriptions, and communication barriers between patients and healthcare providers (5, 11). In Iran, the Ministry of Health is mandated by the fifth and sixth development plans to implement electronic prescriptions. The initial implementation occurred in 2016 in private physician offices in collaboration with the Tamin-E-Ejtemaei organization. Following this, health insurance agencies such as Salamat and Tamin-E-Ejtemaei expanded the program across provinces as a pilot project until January 2021, when it became mandatory for all outpatients nationwide (13, 14).

Until now, in Iran, there has been limited investigation into the complex aspects of electronic prescriptions from the patient’s perspective. This study investigates patients’ attitudes toward electronic prescription and its impact on their satisfaction levels, the quality of healthcare delivery, and their interactions with healthcare professionals, including doctors and pharmacists. The primary objective of this research is to shed light on patients’ understanding of electronic prescribing and its influence on the quality of care, their interactions with prescribers and pharmacists, as well as their perceptions of the benefits and drawbacks of electronic prescribing within the city of Shiraz.

## Materials and methods

This qualitative study is based on one-on-one interviews. Semi-structured and individual interviews were conducted with patients referred to selected pharmacies across all 11 districts of Shiraz city. This approach was chosen to provide the necessary flexibility to explore patients’ attitudes. An initial list of pharmacies in Shiraz city was selected as a stratified sample based on the 11 regions to collect patients’ views on electronic prescribing. Announcements were placed in the selected pharmacies, and invitation letters were included in patients’ medicine packages. This allowed patients willing to participate in the study to call the contact number on the invitation letter and arrange the interview time. Patients aged 18 years or older who had at least three visits to a doctor in the past year and were prescribed an electronic prescription were included in this study.

A semi-structured interview was conducted to collect the necessary data to assess patients’ attitudes, ensuring maximum flexibility in capturing patients’ perspectives. The interview evaluated three areas: patients’ understanding of electronic prescriptions, their relationship with the doctor and pharmacist, and their viewpoint on the advantages and disadvantages of electronic prescribing.

Following data collection, rigorous analysis procedures were implemented. All interviews were transcribed verbatim immediately after recording, with researchers concurrently taking detailed notes during the interviews to ensure the accuracy and completeness of the data.

Thematic analysis, a well-established qualitative research method, was conducted for data analysis. The analysis process adhered to a structured six-step approach:

*Familiarization with the Data*: Researchers immersed themselves in the collected data, gaining a profound understanding of the content and identifying underlying concepts.

*Generation of Initial Codes*: Each concept, including its primary and sub-elements, was systematically assigned a code, facilitating data organization.

*Category Exploration*: Through iterative examination, categories were developed to group related codes, allowing for the identification of overarching themes within the dataset.

*Review of Main and Subcategories*: This critical step involved revisiting codes, categories, and subcategories to ensure they accurately reflected the dataset’s nuances.

*Definition and Naming of Categories and Subcategories*: Distinct definitions and appropriate labels were assigned to each category and subcategory, ensuring clarity and consistency in the analysis.

*Report Preparation*: The final phase involved summarizing and presenting the findings coherently and comprehensively.

The researcher conducted the interviews in a room within the pharmacies. The interviews were recorded, and verbatim transcription was performed after obtaining consent from the interviewees. The transcripts were then cross-verified against the audio recordings. Limited demographic data were also collected as part of the interview process.

Two researchers read and coded transcripts separately. They then discussed the transcripts to identify inconsistencies and reach a consensus on coding decisions.

In the initial stage, both researchers shared common opinions on approximately 76% of the codes. Subsequently, the two researchers re-coded the transcripts, and in the second phase of the review, the agreement on codes reached 99%. The remaining 1% was discussed in a second session, and ultimately, both researchers reached a consensus.

The interviews yielded three main categories and 28 sub-categories. Qualitative analysis was performed using MAXQDA software (version 10), and descriptive statistics for demographic data were calculated using SPSS version 19.

The interview questions were selected from the article titled “Patient perceptions of e-prescribing and its impact on their relationships with providers: A qualitative analysis” (7).

To ensure the validity and accuracy of the qualitative data, the research adhered to Guba and Lincoln’s criteria, which encompass reliability, variability, dependability, and confirmability (15). The interview analysis was conducted iteratively, and the text was shared with participants to rectify potential errors. Various coding methods were also utilized, and an expert in qualitative studies assisted in the analysis.

The present study received approval from the Ethics Committee of Shiraz University of Medical Sciences, Shiraz, Iran (IR.SUMS.REC.1401.361). After securing the necessary permits from the Research Vice-Chancellor of the Faculty of Medical Information and Management and a letter of approval from Shiraz University of Medical Sciences, the researchers explained the research objectives to the participants and introduced themselves. They assured the participants that all recorded information would remain confidential. After that, participants willing to participate in the study were selected, and they were also assured that they could withdraw at any stage of the interview process. Other ethical considerations included: (1) obtaining written consent from the participants, (2) assuring the participants that the study results would be made available to them if they wished, (3) observing ethical considerations in terms of data confidentiality, (4) expressing gratitude to all the people who cooperated in the research, and (5) obtaining approval from the ethics committee.

## Results

Between December and February 2021, we conducted 21 interviews, each lasting approximately 15 to 60 min. The participants comprised of 48% men and 52% women. Table 1 shows the demographic information of the participants.

**Table 1:** Demographic information of interviewed patients.

The average age of the participants	Male	37 ± 10
	Female	33 ± 10
	Total average	35 ± 10
	Diploma and below (*n*) percent	33% (7)
	Master’s degree and bachelor’s degree (*n*) percent	52% (11)
Level of education	Master’s degree and doctorate (*n*) percent	10% (2)
	PhD (*n*) percent	5% (1)
The number of visits to the doctor in the past year	Minimum	3
	Maximum	8
	Average	5 ± 1

Subjects regarding patients’ attitudes toward electronic prescribing were organized into 3 main categories and 11 sub-categories. Some of these sub-categories were further divided into sub-sub-categories. In total, 42 main codes were extracted. Table 2 reveals the detailed breakdown of these categories, sub-categories, and codes.

**Table 2:** Main category, sub-category, sub-sub-category, and main codes extracted from the study.

The main category	Subcategory	Sub subcategory	Original code
Patients’ awareness of the plan	Design definition	——	Correct and accurate definition
			General and imprecise definition
	How to find out when the plan starts	——	Knowing the exact start time of the project
			Knowledge of the starting time of the project with a difference of 3 months
			Knowledge of the starting time of the project with a difference of 6 months
			Knowing the start time of the plan with more than 6 months’ difference
	How to know about the start of the project	——	Through social media
			By visiting a doctor’s office or pharmacy
			Through virtual space
			Other ways (hearing from friends and acquaintances)
Attitude toward prescription and electronic prescription	Knowledge of prescribed services (number and type of medicinal items or diagnostic and therapeutic service) by the doctor	——	Full knowledge and information
			Knowledge and relative information
			Lack of information on the type of prescription
	Information about the prescribed prescription and how to follow up to receive the prescription	Yes (how to find out)	Receive SMS after registration
			Receive the tracking code of the registered prescription from the doctor or his secretary
		does not have	——
	Doctor–patient communication	——	It is better than before
			It has not changed
			It is worse than before
	Duration of service in the pharmacy	——	Earlier than before
			It has not changed
			Later than before
	(pharmacy technical officer) communication with the patient	——	It is better than before
			It has not changed
			It is worse than before
Advantages and disadvantages of electronic prescribing	General view of the plan	——	Positive
			Indifferent
			Negative
	Positive attitude and experiences	Quality and safety	No mistakes in reading version and screw version
			Removal of pen corrosion and bad handwriting
			Not losing the copy
		Reduce costs	Saving paper
		Ease of use	No need to renew the notebook
			Reducing the time of providing some services
			No need to carry paper and notebooks
			Virtual visit and drug registration
			The possibility of receiving from several pharmacies
	Negative attitudes and experiences	Poor infrastructure	Network problems
			Hardware and software problems
			Slow or frequent system crashes
		Time-consuming	Prescription error and returning the prescription to the doctor
		Reduced communication	Less communication between the doctor and the patient
			Failure of doctor and pharmacist to pay attention to the patient
		Reducing the amount of information about the prescription	Not knowing the type of medicine prescribed
			Not knowing the brand of prescribed drugs

The key findings from the analysis are outlined below. It is worth noting that the number of participants may not always be 21 in certain instances, as responses were not mutually exclusive. In certain cases, participants expressed more than one opinion on a specific topic, resulting in a frequency count exceeding 21 for some measures.

### A: patients’ awareness of electronic prescribing

Most participants were not entirely familiar with electronic prescribing, and when asked to provide a detailed explanation, they could not explain what it meant to them. Most explained that the prescriber uses a computer, and the prescription is sent directly to the pharmacy, bypassing manual delivery (*n* = 16).

One of the patients perceived electronic prescriptions as the use of Internet platforms such as WhatsApp for doctor consultations:

“Yes, I know that it is online instead of in person. We call the doctor, and the doctor consults us through WhatsApp, explains what to do, prescribes our medicine, and then we go to the pharmacy to get our medicine.” (P4-ph.A-D1)^1^

Another participant equated it with a person’s authentication system:

“I will provide a national code so that they can identify us. This is referred to as electronic prescription.” (P6-ph.B-D3)

Patients with very limited knowledge of the electronic prescribing system were provided with explanations about these services. Interviews were conducted only after patients had gained a clear understanding of the electronic prescribing system.

These interviews occurred approximately a year after the electronic prescription project was launched in Iran. Over half of the participants (*n* = 13) were unaware of the exact start time of electronic prescriptions. Regarding their awareness of this project, participants learned about it during a doctor’s appointment (*n* = 9), through social media (*n* = 6), and via online platforms (*n* = 3). Additionally, three participants were informed about the project through other means, such as acquaintances and friends.

Regarding the time and method of becoming acquainted with the system, one participant mentioned:

“I believe I learned about it about two years ago or less…through my colleagues.” (P10-ph.C-D9)

Other participants mentioned that they became aware of this program approximately 3 months ago through visiting a pharmacy or a physician:

“I guess it was around two or three months ago that I went to a pharmacy and found out about this.” (P14-ph.D-D5)

“It has been three or four months now… I found out through the media and after visiting the doctor.” (P12-ph. E-D8)

Another participant had detailed information about the beginning of the project:

“It has been electronic for about a year … they announced it on TV.” (P16-ph. F-D6)

### B. attitudes toward electronic prescribing

#### B-1: attitude toward electronic prescribing (in the physician’s office)

Most participants were unaware of the type of services provided by the doctor and the type and number of prescribed medicinal items (*n* = 18). On the other hand, almost half of the participants (*n* = 9) did not perceive any difference in the doctor’s behavior during their visit compared to the previous visits. The second phase of this issue pertained to the group that had experienced negative feelings about their doctor (*n* = 7). Only three participants mentioned that the conditions were better than in the past.

In this regard, one participant mentioned:

“No; They do not disclose the number and type of medicine unless the doctor has the ethics to do so, and they also do not explain.” (P5-ph. G-D11)

Another participant expressed dissatisfaction with the change in doctor–patient communication due to electronic prescribing:

“The relationship has worsened. Doctors used to communicate, but now most doctors are more connected to the system, trying to find the medicine and write the prescription. They used to communicate more than now”.(P7-ph. F-D6)

One participant expressed concerns regarding the electronic prescription being managed by the doctor’s secretary and noted the absence of noticeable changes in the doctor’s behavior compared to the manual prescription writing:

“I've visited a doctor several times since electronic prescriptions were introduced. This doctor did not directly enter medicine details into the system. Instead, he prescribed paper and instructed me to take it to his secretary for typing. It feels like he's still prescribing it as if it were a paper prescription.”(P1-ph. H-D4)

On the other hand, another participant felt satisfied about the type of prescriptions and improved communication with the doctor.

“Every time I visit the doctor, who is familiar with me, he always inquires whether I have certain medicines at home to avoid prescribing them again. When he writes the prescription, he consults us, and since the doctor knows us, he asks about the medicines we already have. The doctor’s clear explanation of my prescription alleviates my concerns.” (P19-ph. I-D2)

#### B-2: attitude toward the electronic prescription (in the pharmacy)

The participants were asked two questions during a visit to the pharmacy to receive their medicine. The first question was about the waiting time to receive the medicine, and the second was about communicating with the pharmacist regarding the necessary explanations of prescribed medicines.

Regarding the waiting time to receive medicine, a total of 17 individuals responded. Nine individuals reported a delay in the delivery services, five reported no difference, and three reported quicker delivery services in the pharmacy.

One patient expressed dissatisfaction with the delayed delivery of the medicine:

“Now there is more of a delay. We used to wait less, but now we have to wait longer. They have sent letters everywhere about the electronic system; we have to wait longer because of the electronic system”(P8-ph.C-D9)

One participant attributed the longer waiting time in the pharmacy to the perceived dishonesty of the pharmacy staff and a lack of patient information:

“The duration of receiving medicine has increased. Sometimes pharmacists lie that the system is not working in order to rest for a while.”(P17-ph. G-D111)

On the other hand, some patients evaluated the delivery time as favorable:

“Now it is faster than the paper prescription.”(P1-ph. H-D4)

Regarding the pharmacy technician’s explanations of the drugs and the pharmacist’s interaction with the patient, seven individuals rated the conditions as worse than before, nine rated them as unchanged from before, and five rated them as improved compared to before. One participant shared their experience with the pharmaceutical manufacturer as follows:

“Yes, The pharmacists inform patients, for instance, that out of this prescription, we do not have two of the medications, or that we offer the Iranian brand or the foreign brand.”(P21-ph. J-D7)

Another participant said:

“Yes. In the pharmacy, they explain the medication, how to take each one, or whether it is a foreign or Iranian brand.”(P8-ph.C-D9)

Yet another patient evaluated the situation as worse than before:

“No, they do not say how many medicines are there. If they do not have a medicine, sometimes they mention that they do not and refer us to check other pharmacies, but sometimes they do not say anything.”(P20-ph.B-D3)

### C: advantages and disadvantages of electronic prescription

#### C-1: positive attitudes and experiences

Patients’ positive attitudes and experiences with electronic prescribing were primarily related to ease of use (*n* = 17), safety and quality (*n* = 9), and cost (*n* = 4). Some individuals reported more than one positive experience, the frequency of which was mentioned in both sections.

The ease of electronic prescription refers to the elimination of the need to renew health insurance booklets, reducing the time spent providing some services, no need to carry a health insurance booklet, and the possibility of receiving single-prescription drugs from several different pharmacies without removing the paper from the insurance booklet.

One of the patients stated:

“The pharmacies used to say that we have one of the drugs, we do not have the other one, and you have to buy the one we do not have without insurance coverage. Previously, there were mistakes in the doctor's handwriting, or the doctor had stamped only one medicine, and the other was not stamped. Now these problems have been solved.” (P5-ph. G-D11)

Issues regarding safety and quality include reducing medication errors, increasing access to information for prescribers, and avoiding losing prescriptions.

A participant mentioned in this regard that:

“The most important advantage, in my opinion, is that the mistakes that pharmacies and lab technicians used to make because of doctors' bad handwriting will not be repeated. Secondly, patients used to lose prescriptions. Before, if the doctor wrote the prescription incorrectly or it was in poor handwriting, we had to go back to the doctor to correct it. But now, we no longer have to return to the doctor because they write prescriptions with a computer. It is always written correctly and is no longer a problem.”(P5-ph. G-D11)

Positive experiences have been reported in terms of both overall cost reduction for the healthcare system and environmental protection.

A 23-year-old woman made the following positive observations:

“For example, I believe that less paper should be used. I am one of those who believe that life should be green. The less paper we use, the less environmental damage there is. I think this is a very positive thing.”(P19-ph. I-D2)

#### C-2: attitude and negative experiences

Patients’ negative perceptions and experiences of electronic prescribing predominantly point to the infrastructural problems, the slowness and uncertainty of the system (*n* = 18), a feeling of less control over their prescriptions (*n* = 18), communication problems with prescribers (*n* = 13), and errors in the timing of prescriptions by their doctor (*n* = 10).

Several patients reported that the doctor incorrectly prescribed their electronic prescription.

Communication challenges with prescribers include worsening interpersonal communication, as the prescriber seemed to be more focused on the computer than interacting with the patient. Communication challenges with pharmacists included missing the opportunity to interact at the prescription delivery stage.

In general, patients associate electronic prescribing with a loss of control over their prescriptions. On the other hand, delays in sending prescriptions lead to delays in receiving drugs.

Patients also reported that previous written prescriptions provided them with personal access to information about what was being prescribed, even if it was just the name of the drug.

Two patient statements presented below are examples of negative perceptions/experiences of electronic prescription:

“A 35-year-old woman said: My uncle's daughter once went to the pharmacy. She was allergic to a certain medicine, and the doctor mistakenly prescribed that medicine. Luckily, the pharmacist, who knew my cousin well, realized she was allergic to the prescribed medicine. The pharmacist asked her, “Don't you have an allergy? Why do you want to take this medicine? Her physician had already changed that medicine for her. If she had taken the wrong medicine that the doctor had prescribed for her, it would have been very dangerous.”(P8-ph.C-D9)

Another patient said:

“I asked several times and from different pharmacies about the medicine and why it was given to me. I realized that it had nothing to do with my medicine and nothing to do with my disease. When I went back to the doctor and questioned it, he said that he had typed the drug code wrongly. He then rewrote the prescription, and I had to leave and come back again. It is true that there were mistakes in reading the prescription, and now those mistakes are not happening. Still, there could be a problem with the medicine code due to doctors’ lack of familiarity with this new system. Some doctors do not have complete information about the new system, so they cannot work with it properly and prescribe the wrong medicine. The relationship has unfortunately deteriorated. Previously, doctors would communicate, but now it seems that most doctors primarily concentrate on navigating the system to locate the medication and write the prescription. The level of communication was notably higher in the past than the present.”(P7-ph. F-D6)

Another participant stated:

“The disadvantages that I would say are internet and website outages, and patient delays. It means that there is internet, but the site might have a problem. More time is being spent.”(P14-ph.D-D5)

Despite participants identifying both advantages and disadvantages of electronic prescription, some patients reported no personal impact from the technology or expressed neutral opinions about its use. Specifically, patients did not report any changes in communication with the doctor (*n* = 9), communication with the pharmacist (*n* = 5), or the duration of service in the pharmacy (*n* = 5).

## Discussion

This study aimed to explore patients’ attitudes toward electronic prescription systems in Shiraz. Interviews were conducted with 21 patients who sought medical services and visited pharmacies across 11 city districts for medication. The findings revealed a range of positive and negative attitudes and experiences among patients.

Patients in our study reported positive attitudes and experiences regarding electronic prescribing. They emphasized the ease of use, enhanced safety, improved healthcare quality, and cost reduction associated with this system. Seventeen patients mentioned a positive experience, and one only mentioned positive points. These findings align with a study conducted in Poland in 2021, which also highlighted the convenience of electronic prescribing, the reduced risk of prescription loss, and the elimination of the need for in-person doctor visits (14). On the other hand, patients in our survey spoke negatively about their experiences and views related to infrastructural difficulties, system hiccups, slowness, electronic prescription mistakes, feeling like they have less control over their prescriptions, and poor contact with their prescribers. Eighteen patients mentioned at least one negative point, and three people mentioned only negative points. These findings correspond with those of a previous article (9). Interestingly, our research suggests that patients generally held a more favorable opinion of electronic prescribing compared to the perspectives of doctors and pharmacists, as noted in a study conducted by Amlashi et al. in 1401 (equivalent to 2022–2023 in the Persian calendar) (16).

In general, patients were unfamiliar with electronic prescribing, and they felt that using this technology had little impact on their care. However, patients reported positive attitudes and experiences regarding ease of use, safety, quality, and cost. The participants in this study were unaware of the capabilities of electronic prescribing, such as checking the records of previous prescriptions and utilizing machine learning methods to help the doctor improve the quality of care and reduce the incidence of errors. This issue is addressed by a study named “Patient perceptions of e-prescribing and its impact on their relationships with providers: a qualitative analysis” (7). On the other hand, the results of this study differed from the results of a study titled “Patient perception and satisfaction with the electronic prescription system: results of the PERSA-RE questionnaire” (4).

The study highlighted the challenges stemming from the limitations of the e-prescribing system, which resulted in an increased workload and time consumption for patients. These limitations reduced the effectiveness of e-prescribing, ultimately forcing patients to obtain only a portion of their prescribed medications. Consequently, patients were compelled to cover the costs of medications not covered by their insurance plans, contributing to a financial burden. These findings align with the results of a study titled “A Pilot Study to Evaluate Prescription Transfer and Drug Collection through a New Electronic Prescription Service: A Cross-Sectional Survey” conducted in Saudi Arabia (12). This correspondence underscores the universal nature of the challenges associated with e-prescribing system limitations and their impacts on patient care and financial well-being.

There is still hope that the passage of time and the usage of electronic prescriptions will enhance patient’s experiences and knowledge about all of their features. Despite the disadvantages of electronic prescribing, some patients have provided valuable suggestions to improve their conditions. In a study evaluating prescription transfer and drug collection through a new electronic prescription service (12), the participants were not interested in making suggestions. However, most participants in this study were satisfied with the plan’s encouragement and presented significant suggestions. Their suggestions included using a printer to print prescriptions if requested by the patient, sending the contents of registered prescriptions to the patient’s mobile number, creating a proper hardware infrastructure, adapting the doctor–patient interaction in response to the changes in the platform of interactions, and providing 24-h support from the technical team to remove existing obstacles. These were some of the proposals mentioned by patients to resolve problems and improve the existing situation. This study’s results were inconsistent with the study conducted in Saudi Arabia (12).

In addition to the common concerns that patients express about losing opportunities to interact with the doctor, the non-compliance of the pharmacy and the pharmacist during the prescription delivery phase was also a point of worry. Some patients were concerned that because they did not know the content of the prescribed medication, they may be delivered more or less medicine, or without their knowledge, a specific type and brand of medicine that the doctor intended may not be delivered to them. Participants suggested that information at the time of prescription, such as printed patient information and post-visit summaries, could be made available to these people to address such concerns. In their study, Jabraeili et al. suggested that system developers should improve their capabilities by properly communicating with users and fully understanding their real needs, which is consistent with the suggestions made by the participants in this study (17).

Most global studies have examined the technical advantages and disadvantages of electronic prescribing systems. These studies have focused on the attitudes of doctors, pharmacists, and other personnel related to electronic prescribing, with few studies conducted on patients’ attitudes toward electronic prescribing (5, 11, 13). Like other countries, following the introduction of electronic prescriptions in Iran, studies have been conducted to assess their advantages, disadvantages, and problems, particularly from the technical perspectives of doctors and pharmacists. However, no research has been conducted regarding the patients’ attitudes toward this issue (2, 3). Patients and those who refer to health and treatment centers for medical services can be important beneficiaries of the electronic prescribing system. Patient satisfaction with the electronic prescribing system will help patients adhere to treatment with better and more effective communication with the doctor. It is very important to know the strengths and weaknesses from the perspective of patients, who are the significant beneficiaries of this plan (2, 5). However, physicians and pharmacists should also be aware of the potential problems that can arise from miscommunication related to electronic prescribing. More research is needed to determine how clinicians can use these existing tools to improve patient education and prescription decisions.

This study had several limitations. One of these limitations was the small sample size. Another limitation was the generalizability of the results. Although qualitative studies inherently have limited generalizability, an effort was made to increase the generalizability of the results by including women with different characteristics. Another limitation was the relative youth of the interviewed population compared to other studies. This issue is due to the better understanding of this group of interviewees regarding the use of emerging technologies, including electronic prescriptions. This group of patients was also more willing to answer our questions. Despite these limitations, the study provides valuable insights into patients’ attitudes toward electronic prescriptions. Further research with a larger and more diverse sample size could help to address these limitations.

Given the importance of patient–therapist communication in healthcare, it is essential to explore the changing dynamics of doctor–patient interactions in the context of electronic prescribing. Future research can delve into the nature of these evolving communication patterns and aim to develop strategies to mitigate potential harm. Such research would illuminate ways to optimize the doctor–patient relationship within the framework of electronic prescribing. Moreover, addressing patients’ concerns about privacy violations is a pressing issue. Future studies should focus on implementing measures to alleviate patient apprehension regarding the security and confidentiality of their health information in electronic prescribing systems. By enhancing data security and privacy safeguards, healthcare providers can foster greater patient trust and confidence, ultimately improving the adoption and acceptance of electronic prescribing technologies. Given that the mean age of the statistical sample in our study was 35 ± 10, extrapolating the findings of this study to communities with a different mean age requires careful consideration.

## Conclusion

Patients reported positive attitudes and experiences regarding the ease of use, safety, quality, and cost of electronic prescribing. However, they also reported negative attitudes and experiences related to infrastructural problems, system delays and interruptions, errors in electronic prescribing, a perceived loss of control over their prescriptions, and communication problems with their prescribers. Many patients’ concerns stemmed from a lack of knowledge about the program and its advantages. Therefore, educating medical staff, especially doctors and pharmacists, is necessary to adapt their interactions to the electronic prescribing system. This includes familiarizing them with more features of electronic prescription to improve their use. By doing so, we can address patients’ concerns and enhance their experience with electronic prescribing.

## Data availability statement

The raw data supporting the conclusions of this article will be made available by the authors, without undue reservation.

## Ethics statement

The studies involving humans were approved by the Ethics committee of Shiraz University of Medical Sciences, Shiraz, Iran (IR.SUMS.REC.1401.361). The studies were conducted in accordance with the local legislation and institutional requirements. The participants provided their written informed consent to participate in this study.

## Author contributions

SA: Data curation, Conceptualization, Methodology, Formal analysis, validation, Writing – original draft. SZ: Conceptualization, Methodology, validation, supervision, Project administration, Writing – original draft, Writing – review & editing. MR: Conceptualization, Methodology, Investigation, Writing – original draft.

## Footnotes

^1^P: Patricipant/ph: pharmacy/D: district.
